# Schistosomiasis is more prevalent than previously thought: what does it mean for public health goals, policies, strategies, guidelines and intervention programs?

**DOI:** 10.1186/s40249-017-0275-5

**Published:** 2017-03-22

**Authors:** Daniel G. Colley, Tamara S. Andros, Carl H. Campbell

**Affiliations:** 1 Schistosomiasis Consortium for Operational Research and Evaluation (SCORE), Center for Tropical and Emerging Global Diseases (CTEGD), University of Georgia, 500 DW Brooks Drive, Room 330B Coverdell Center, Athens, Georgia 30602 USA; 2 Department of Microbiology, University of Georgia, Athens, Georgia 30602 USA

**Keywords:** Schistosomiasis mansoni, Circulating cathodic antigen, Kato-Katz, Mapping, Diagnosis

## Abstract

Mapping and diagnosis of infections by the three major schistosome species (*Schistosoma haematobium*, *S. mansoni* and *S. japonicum*) has been done with assays that are known to be specific but increasingly insensitive as prevalence declines or in areas with already low prevalence of infection. This becomes a true challenge to achieving the goal of elimination of schistosomiasis because the multiplicative portion of the life-cycle of schistosomes, in the snail vector, favors continued transmission as long as even a few people maintain low numbers of worms that pass eggs in their excreta. New mapping tools based on detection of worm antigens (circulating cathodic antigen – CCA; circulating anodic antigen – CAA) in urine of those infected are highly sensitive and the CAA assay is reported to be highly specific. Using these tools in areas of low prevalence of all three of these species of schistosomes has demonstrated that more people harbor adult worms than are regularly excreting eggs at a level detectable by the usual stool assay (Kato-Katz) or by urine filtration. In very low prevalence areas this is sometimes 6- to10-fold more. Faced with what appears to be a sizable population of “egg-negative/worm-positive schistosomiasis” especially in areas of very low prevalence, national NTD programs are confounded about what guidelines and strategies they should enact if they are to proceed toward a goal of elimination. There is a critical need for continued evaluation of the assays involved and to understand the contribution of this “egg-negative/worm-positive schistosomiasis” condition to both individual morbidity and community transmission. There is also a critical need for new guidelines based on the use of these more sensitive assays for those national NTD programs that wish to move forward to strategies designed for elimination.

**Electronic supplementary material:**

The online version of this article (doi:10.1186/s40249-017-0275-5) contains supplementary material, which is available to authorized users.

## Multilingual abstracts

Please see Additional file 1 for translations of the abstract into the five official working languages of the United Nations.

## Background

Historically, schistosomiasis has been diagnosed and mapped by multiple different methods that have relied on microscopy of excreta [1–5] for schistosome eggs, detection of antibodies against schistosome antigens [6–8], viable parasitological assays such as egg hatching from excreta [9] or frank signs of morbidity [10]. Most commonly, infection by *Schistosoma haematobium* has been mapped using questionnaires, gross hematuria, micro-hematuria (by hemastix) or urine filtration followed by microscopy for *S. haematobium* eggs [3–5, 10]. *S. mansoni* and *S. japonicum*, however, are most commonly mapped by Kato-Katz (KK) stool microscopy [1] or some other stool concentration technique [5, 11] to observe schistosome eggs. These assays have been entirely satisfactory for programs focused on bringing high levels of prevalence and intensity of infection down to “manageable” points where severe morbidity is taken to a minimum and subtle morbidities lessened [12, 13]. However, based on World Health Assembly Resolution 54.19 in 2001, purchases and donations of praziquantel (PZQ) and enabling organizations such as the Schistosomiasis Control Initiative and others, many countries have implemented Mass Drug Administration (MDA) programs leading to lower countrywide prevalence and intensity levels of schistosomiasis. Some countries, such as Japan, China and Egypt have led the way with 50-60 yearlong programs to achieve this end. All of this has led to the World Health Assembly (WHA) Resolutions 65.21 and 66.12, in 2012 and 2013, respectively, and the London Declaration on Neglected Tropical Diseases (NTDs) in 2012 [14]. The WHA resolutions and the subsequent WHO/NTD Road Map in 2011 [15] now seek to evolve, where feasible, the programmatic focus from morbidity control to elimination of schistosomiasis as a public health problem and eventually to elimination of transmission. The London Declaration assists this shift in goals through donations by companies and government and non-government agencies. In regard to schistosomiasis, this is clearly manifested by the major donation by Merck-KGaA of praziquantel (PZQ) for morbidity control and elimination. A real shift in emphasis to these goals of elimination will require substantial changes based on comprehensively knowing and following the on-the-ground situation in regard to the prevalence and intensity of human schistosome infections, coupled with conscious and meaningful changes in public health program strategies and tools. Although there is no “gold standard” for detecting schistosome infections, that knowledge and those strategies and tools will need to be driven by mapping and diagnostic assays that are more sensitive than what have served the programs well in the past. Those goals will also need to be founded on new guidelines and the implementation of new policies predicated on the use of the more sensitive assays. This commentary will focus primarily on the currently relevant case of mapping of *S. mansoni* infection by the use of the commercially available urine Point-of-care Circulating Cathodic Antigen (POC-CCA) assay in comparison with the stool KK assay and discuss the challenges and conundrums raised in changing to more sensitive assays, and the guidelines and strategies needed for moving towards elimination of schistosomiasis.

### An available urine assay for mapping *S. mansoni* infections – challenges and opportunities

Based on detection of the Circulating Cathodic Antigen (CCA), first reported on in the mid-1970s [16–18], the commercially available POC-CCA cassette assay to detect CCA from *S. mansoni* worms in urine became available in 2008. Its major advantages are the use of urine rather than stool samples, the ability to do the assay either on-the-spot or on stored urines, and that the assay does not require a microscope and highly trained microscopists. These characteristics have provided an impetus to utilize this assay for the mapping of *S. mansoni* in many studies [19] and now some national programs. The drawbacks of using this assay compared to the KK stool assay are that 1) it does not detect Soil-transmitted Helminth (STH) eggs, 2) does not provide a quantified number of eggs per gram of stool, and 3) presents a challenge regarding the uniform interpretation of “trace” readings.

The limitation in quantification of eggs is somewhat mitigated because the density of the band observed in a positive test bears some relationship to assessment of eggs per gram by KK [19]. Also, electronic POC-CCA readers can assist in quantifying the bands [20] (personal observation, Carl H. Campbell, Jr.).

Of more concern is the assessment of what constitutes a “Trace” reading. The manufacturer maintains that a “trace” (i.e., a faint band) should be considered a positive, but data from multiple sites and comparisons conducted in Kenya suggest that different people do or do not see light bands in the assay. In addition, when some investigators see a faint band they have chosen to refer to this as “trace-negative” and dealt with it as a negative assay in their analyses. However, in areas of low prevalence and intensity even if trace readings are considered negative the prevalence by POC-CCA is uniformly higher that observed by KK [19, 21]. In this commentary we will refer to faint bands (trace readings) as positive.

Unfortunately, although both *S. mansoni* and *S. haematobium* produce CCA, the POC-CCA assay is less reliable in determining *S. haematobium* infection [22, 23]. This may be due to different quantities of CCA produced by each species or perhaps differences in the CCA from each species in terms of clearance and urine concentrations. Interestingly, one proof-of-concept report indicates that the POC-CCA assay can be used to detect *S. mekongi* and *S. japonicum* infections at reasonably high level of specificity, albeit at similar levels of sensitivity as a Kato-Katz assay [24]. A second publication focused on the detection of Circulating Anodic Antigen (CAA) for detection of *S. japonicum* in low prevalence areas of China indicated that the POC-CCA assay did not show sufficient sensitivity to be useful in this setting [25].

### Using the POC-CCA to map *S. mansoni* infections leads to new insights about prevalence, causes us to confront new challenges and provides fresh opportunities

Based on multiple studies it has become clear that when the prevalence by KK stool assay is below 50% the POC-CCA assay detects many more *S. mansoni* infections [19]. When the egg-determined prevalence by KK is very low (below 20%) the evidence from multiple studies indicates that the worm-determined prevalence by POC-CCA is often 3- to 6-fold higher [19]. This constitutes a major challenge for national programs, because if they use prevalence cutoffs from the current WHO guidelines to dictate the appropriate programmatic response, they must conduct much more extensive and frequent MDA than if they relied on KK prevalence; however, because guidance incorporating experience with POC-CCA has not been issued, programs are uncertain whether or not to do so.

In settings of low KK prevalence, many (sometimes >50%) of the POC-CCA positives are due to trace readings, which are unlikely to indicate moderate or high-intensity infections. Furthermore, it is unknown whether some or many of the trace readings represent false positives. To try to address this question in the situation presented by schistosomiasis, i.e., without a gold standard, is challenging. KK results cannot be used as a comparator, because they are known to be insensitive in these circumstances. Several efforts have been made to determine the false positive rate. Those assessments used the POC-CCA in non-schistosomiasis endemic areas, where any positive results likely represents false-positives (Ecuador and non-endemic areas of Ethiopia). In those evaluations, there was 1 trace reading out of 243 children, many of these children had STHs but lived in schistosomiasis non-endemic areas [26, 27].

Another approach is to use a laboratory-based assay for Circulating Anodic Antigen (CAA) (which is also a product of schistosome worms), which is thought to be even more sensitive and more specific than the POC-CCA assay [16, 18, 28] and that can then be used to examine urines from people who are egg-negative but CCA-positive. Such studies have been and are being done for *S. mansoni*, but are not yet published (personal observation, Daniel Colley). Using the Up-converting Phosphor (UCP)-CAA assay as a “confirmatory” test the countrywide prevalence obtained by testing a subsample of the urines generally yields levels of prevalence that are 67 and 87% of what was seen based on the POC-CCA cassette assay. Thus, using the UCP-CAA assay as the best available standard, the indication is that mapping with the POC-CCA assay over-estimates the prevalence by ~ 15 to 30% (personal observation, Daniel Colley and Carl Campbell). Almost all readings of the POC-CCA assay of 1+ and above correlate strongly with egg-positivity, and thus provide solid antigenic evidence of viable worms. This, however, still leaves a large proportion of the population being mapped as positive for having living worms in their blood vessels, but without detectable schistosome eggs in their stools on any given day. The daily variability of the urine POC-CCA assay is substantially less than the variability of the KK stool assay in detecting infection [26, 29, 30].

When there is no gold standard for diagnosis, another way to compare the relative accuracy of two or more different assays is to estimate their most likely sensitivities and specificities using a statistical approach called Latent Class Analysis (LCA) [31–33]. The LCA uses information from all tests results, then estimates a ‘true’ prevalence of infection and calculates the most likely sensitivity and specificity values for each competing test. This can be done only if two or more different assays are recorded for the same person each time. While the LCA may underestimate the specificity of an assay that is highly sensitive relative to its comparators, it provides a more realistic estimate of an assay’s diagnostic performance relative to an unmeasured true infection status. By LCA, the POC-CCA assay is consistently much more sensitive than KK. Using LCA, the estimated specificity of the POC-CCA is somewhat lower than for KK because the finding of an egg is presumed to make the KK 100% specific [27, 34–36]. In terms of test sensitivity, however, a KK performed on one or even three daily stools is known to provide only mediocre detection of lightly infected subjects [37, 38]. This means that it is in areas with very low prevalence (by KK) that the higher sensitivity of the POC-CCA assay renders it most valuable in terms of making programmatic decisions about prevalence and in planning future interventions. As further comparative studies are done with the KK, POC-CCA, PCR [27], UCP-CAA, and antibody, assays, it will be of great interest to evaluate these in parallel by LCA to compare their performance and then find their optimal use in schistosomiasis control programs.

### What does it mean to find people (sometimes many people) with low (or no) eggs by KK stool assays that have schistosomes by detectable CCA?

It must always be remembered that these two assays detect different life-cycle stages – the eggs and the worms – and that it is possible to have worms without eggs, but not possible to have excretion of eggs without worms (after a suitable time period for egg excretion following effective anti-worm treatment, usually thought to be ~ 2–3 weeks for viable eggs and 4–6 weeks for dead eggs). Countries or villages where most people have very low intensities of infection by egg detection yet a substantial proportion of those same individuals have detectable CCA in their urine face a difficult programmatic decision. Part of the challenge of this situation is the lack of current guidelines related to newer assays. In part, this is related to a lack of sufficient data to allow a full understanding of what this newly identified situation actually means. Also, it is due, in part, to a lack of clarity regarding the programmatic goals, i.e., the programmatic decision of whether to continue to provide MDA to populations with very, very low worm burdens may be different in programs still controlling morbidity as compared to those seeking to achieve elimination. Such decisions may also need to differ in different areas of a given country.

Particularly as communities move towards elimination of schistosomiasis based on KK or urine filtration, understanding the implications of “egg-negative schistosomiasis” is going to be increasingly important. Without a true gold standard for detection of the number or status of worms in such individuals, we will not know what the implications of this state are for the individual or for potential transmission to others. Possible explanations for egg-negative schistosomiasis are described in Table 1. The appropriate response for the individual and for the public health system depends on which of these is correct.

**Table 1:** How can someone be Kato-Katz egg-negative and POC-CCA-positive?

1. The KK is insensitive and missed an egg:
a) the egg was in another part of the stool b) the egg was excreted on a different day
2. The POC-CCA is not sufficiently specific: Because the anti-CCA mAb reacted with the CCA epitope produced in another situation (Pregnancy, Infection, Tumors, Autoimmune diseases, etc.)(most studies have been done in children)
3. The POC-CCA readers/technicians were insufficiently trained or trained differently in different programs/teams; and trace readings are difficult to determine
4. Temporary suppression of egg-production by worms damaged, but not killed by praziquantel treatment
5. The female worms went through menopause
6. All immature or stunted female infections
7. All male infections
8. Anti-fecundity immunity reduced or stopped egg production

There is ample evidence for no.1 in Table 1, i.e., that the KK stool exam is insensitive in low intensity infections and that this can be due to intermittent egg excretion [30, 39, 40] and/or a sampling error due to the uneven distribution of eggs in a given stool sample [2]. The data thus far regarding no. 2 in Table 1 are that the anti-CCA monoclonal antibody used in the POC-CCA assay is quite specific. However, most of the use of the monoclonal in the commercial POC-CCA format has been done on children and most of them would not be expected to have widespread alternative causes of cross-reactivity (except other NTDs which have been controlled for [26, 27]). Thus, it is not yet possible to completely rule out some potential cross-reactivity in adults. Number 3 in Table 1 is potentially an explanation for some of the trace POC-CCA readings because of individual visual acuity and/or differential training as to what truly constitutes a trace reading. The trace is, in fact, sometimes challenging to read and could lead to reporting false-positives by some readers, as well as false-positives even when multiple readers agree. The information provided above regarding the use of the “confirmatory” UCP-CAA assay would indicate that this is perhaps a problem with some of trace readings, but not the majority of them. Number 4 in the Table reflects the finding that upon treatment with PZQ some worms are damaged and experience temporary suppression of oviposition but can subsequently rebound and produce viable eggs, even months later [41]. Do female worms go through menopause, or decline significantly in their ability to produce eggs, while remaining long-lived, as hypothesized in no. 5 of Table 1? We simply do not know. Numbers 6 and 7 in Table 1 are both possible, although single-sex female infections do not develop to full maturity. Differential output of CCA by males and females with females making more per worm has been shown in vitro [42], but whether this is true of intravascular dwelling worms is not known. Number 8 is certainly a possibility because there is published epidemiological- and modeling-based evidence for anti-fecundity immunity that would reduce the production of eggs while allowing continued adult worm infections [43, 44]. Also, experimentally adult worm pairs that had ceased to produce eggs in a very chronic baboon infection led to egg excretion when the same worms were transplanted intravascularly into a naïve baboon [45]. Other experimental support for anti-fecundity immunity in chronic schistosome infections comes from the *S. bovis* literature [46, 47] and candidate vaccine literature [48, 49]. Thus the inability to make and excrete eggs in the chronic setting could have been due to some level of anti-fecundity immunity.

### What are the real questions about “egg-negative schistosomiasis” for the development of public health guidelines and the implementation of national programs?

If we take as a given that at low levels of prevalence and intensity “egg-negative schistosomiasis” exists, the real questions are: “What does this mean for such individuals and what are its implications for NTD programs?” Again, in part, the answers to those questions rest on a need for more data to provide a better understanding of why this situation exists and what it means. We posit that “egg-negative schistosomiasis” is most likely to exist because there are relatively few worms and they make eggs either not at all or infrequently (no. 1a & no. 1b in Table 1; possibly due to no. 5 and no. 8 in Table 1). The literature tells us that even in places of higher prevalence and intensity some infected individuals sporadically produce eggs in the stool and the number of eggs per gram of stool varies extensively on a day-to-day basis [30, 39, 40]. Thus it follows that someone with very low worm burdens (trace or 1+ by POC-CCA) may only excrete eggs sporadically. However, it is challenging to prove that this is the case. Perhaps they never produce eggs, or perhaps they produce eggs sporadically, but never excrete them. The latter situation will not be addressable with current tools, but the former (i.e., sporadic production/excretion of eggs by a few worms) can be asked by doing very laborious but important consecutive stool assays (by KK microscopically and by doing Hatch Tests with larger volumes of stool to observe miracidial hatching) over weeks or even months. The information obtained by such studies in very low prevalence and intensity areas will be important for individuals and a control program, and especially for an elimination program. Such extensive, longitudinal stool collections and assays have been done in the past, but in areas with higher prevalence and intensity infections and only up to about 10 days’ time [30, 39, 40].

Why would such data be so important for an infected individual? An individual harboring a few worms that are not producing eggs may not experience health consequences, but if these worms do sporadically produce eggs (whether they are excreted or not) the person may continue to have chronic inflammatory responses to schistosome eggs that are known to cause anemia [50], and therefore they would be at continued risk of morbidity due to schistosomiasis [51–53].

The different implications of no eggs excreted vs. sporadic excretion of eggs by a few worms have perhaps even greater public health implications as national programs move to eliminate schistosomiasis. The level of impact of sporadic excretion of eggs rests on the biology of the life cycle of schistosomes and the goals of the national program involved. The major multiplicative phase of the life cycle of schistosomes takes place within the intermediate host, the snail vector. Thus, one person sporadically passing eggs in their stool and contaminating a fresh water environment in which susceptible host snails exist is sufficient to maintain a continuing life cycle in a community. This, obviously, would not allow the elimination of transmission – or at the very least it would mean there would be a very long time to achieve R_0_ or the nirvana of a “break-point”.

### More sensitive assays always require re-calibration of appropriate guidelines

Understanding “egg-negative schistosomiasis,” especially in low prevalence, low intensity areas, is therefore a primary need, both in regard to individual morbidity and in terms of national programs regarding what and how to move to elimination. Without additional data on the occurrence and/or frequency of egg excretion by those people harboring antigen-producing (CAA and CCA) schistosome worms, the best that can be done is to use the data available. In Burundi [21], Rwanda [54] and Egypt [55], extensive school surveys using KK stool and POC-CCA urine assays indicate that long-term (8 to 25 years) mass drug administration programs with PZQ can achieve national or regional prevalence levels of 1-2% by KK, but that concomitantly in these communities, levels by POC-CCA are much higher (ranging from 10 to 80%). The current WHO guidelines for morbidity control of *S. mansoni* infections, based on prevalence and intensity by KK, are generally not useful in these settings and are certainly not useful for moving towards elimination, nor were they developed for this new goal. We currently do not know the egg excretion potential of those in areas such as Burundi, Rwanda and the Nile delta of Egypt who are egg-negative by KK and positive by trace POC-CCA (egg-/CCA-trace). If the goal of a program is elimination, it is probably prudent to assume that transmission can and will continue if these individuals are not treated. This is because they may be excreting eggs sufficiently, albeit sporadically, to perpetuate transmission. It is simply not known if they are or are not a threat to elimination. Thus, guidance for an elimination program might be to continue annual or more frequent MDAs (or move to test and treat alternatives involving household or work group members) with PZQ and institute other transmission control efforts such as focal snail control and intensive behavior change along with water and sanitation efforts. If the program decides it is not yet ready to move towards elimination, but it needs to continue to focus on morbidity control and to lower prevalence and intensity to a point that elimination can be approached, then the guidance might be to continue with annual MDA in areas of a given moderate POC-CCA prevalence, for example above 20%, until the move toward elimination is decided upon.

A “straw man” proposal when using mapping by POC-CCA prevalence might be:

To gain or maintain control of schistosomiasis: Annual or more frequent MDA is appropriate for any area with >20% by POC-CCA

To move to elimination of schistosomiasis: Variations of test and treat plus “adjunct” means of control in areas with <20% by POC-CCA

To eliminate schistosomiasis transmission: Broad-based test and treat plus “adjunct” means of control in areas with <10% by POC-CCA

To assume elimination of transmission: Broad-based determination of <10% by POC-CCA using an agreed upon mapping strategy and confirmed by subsampling and use of a more sensitive and specific assay at less than 1–2%

To obtain verification and maintain elimination: Broad-based/sentinel site surveillance for human exposure (with POC-CCA or UCP-CAA or detection of antibodies in young children) and/or xenomonitoring (by molecular assays) for at least 3 years (verification) and indefinitely, perhaps on an every other year basis (maintenance of elimination status)

This “straw man” proposal is only that. It is not meant to suggest that these are the correct thresholds or intervention strategies. Perhaps they are, and more likely they are not. It is only intended to provide fuel to initiate discussions in the community of investigators, program managers, public health and funding administrators concerned about where control and elimination of schistosomiasis is headed over the next 3-5 years. It is clear that not all countries, or even all districts/regions of a country, are at the same level in regard to the gaining, maintaining, moving towards elimination, eliminating and maintaining the surveillance needed for elimination. Thus the guidelines need to encompass multiple different epidemiologic situations, levels of political commitment and goals. It is also clear from progress in many different infectious and chronic diseases that as new mapping and diagnostic tools are developed that are more sensitive and specific, the guidelines need to be regularly re-assessed rather than written-in-stone, and re-formulated as needed, to encompass our improved ability to discern the actual situation in the field.

## Conclusion

This is an exciting and an exasperating time for national NTD programs, donors and other stakeholders that deal with control and the potential elimination of schistosomiasis. The excitement is clear. Much of the drug needed is donated, most endemic countries have developed appropriate NTD strategic plans and most have also started MDA programs at some level. A considerable portion of the funding needed for implementation of the plans is available through various agencies, as well as the endemic countries themselves. This is an unprecedented time and the opportunity to truly alleviate some of the world’s most impoverished populations of their schistosomes is here. At the same time, with newer tools and new studies we now know accomplishing the goal of elimination of schistosomiasis will be even more challenging than was anticipated. Current, available tools tell us there is more “out there” than we thought, and the studies reconfirm what those in the schistosomiasis field have known all along, that distribution of the infection is very focal and not all locations respond to control measures equally. Nevertheless, the time is right to push ahead. However, depending upon the current country prevalence levels and the focal distributions of infection, deciding between controlling morbidity or moving towards elimination and surveillance will be difficult decisions. As with any public health program, whether it is to control, eliminate or eradicate, continued research – both basic and operational – is needed throughout the campaign. As a great spokesman for baseball and philosophy once said, “It ain’t over ‘til its over” [56] and that means you will always need new insights and tools and the trick is to be flexible enough and open enough to know how best to use them – until your goal is accomplished.

## Additional file

Additional file 1: Multilingual abstract in the five official working languages of the United Nations. (PDF 552 kb)

## Abbreviations

CAA: Circulating anodic antigen
CCA: Circulating cathodic antigen
KK: Kato-Katz
LCA: Latent class analysis
MDA: Mass drug administration
NTD: Neglected tropical disease
POC: Point-of-care
PZQ: Praziquantel
UCP: Up-converting phosphor
WHA: World health assembly
WHO: World health organization
