# Supplementary material for: Schistosomiasis is more prevalent than previously thought: what does it mean for public health goals, policies, strategies, guidelines and intervention programs?
Source: Infect Dis Poverty. 2017 Mar 22;6:63. doi: 10.1186/s40249-017-0275-5 (PMC5361841; doi:10.1186/s40249-017-0275-5)
Supplement: Additional file 1: — Multilingual abstract in the five official working languages of the United Nations. (PDF 552 kb) [file 40249_2017_275_MOESM1_ESM.pdf]

Translation of the abstract into the six official working languages of the United Nations

البلهارسيا أكثر انتشارا مما كان يعتقد سابقا: ماذا يعني هذا بالنسبة لأهداف الصحة العامة والسياسات والاستراتيجيات والمبادئ التوجيهية وبرامج التدخل؟

دانيال ج. كولي، تمارا س. أندروس وكارل هـ. كامبل، الابن

الملخص

تم خرطنة وتشخيص العدوى لأنواع البلهارسيا الرئيسية الثلاثة (البلهارسيا البولية، البلهارسيا المعوية و البلهارسيات اليابانية) باستخدام المقايسات المعروفة بكونها محددة ولكن غير حساسة على نحو متزايد مع تراجع الانتشار أو في المناطق ذات الانتشار المنخفض بالفعل للعدوى. ويصبح هذا التحدي الحقيقي في تحقيق هدف القضاء على البلهارسيا لأن الجزء المضاعف لدورة حياة البلهارسيا، في ناقلات الحلزون، يفضل استمرار الانتقال طالما حتى في وجود عدد قليل من الناس يحملون عدد قليل من الديدان التي يمر عبرها البيض في فضلاتهم. أدوات الخرطنة الجديدة تقوم على كشف مستضدات الدودة (تعميم المستضد الكاثودي - CCA، تعميم المستضد الأنودي - CCA) في بول المصابين حساسة للغاية وتعميم المستضد الأنودي وجد محددا للغاية. استخدام هذه الأدوات في مناطق الانتشار المنخفض لأنواع البلهارسيا الثلاثة أثبت أن المزيد من الناس يحملون ديدان بالغة تقوم بإخراج البيض بانتظام عند مستوى يمكن كشفه بواسطة فحص البراز المعتاد (كاتو-كاتز) أو عن طريق الترسيب البول. في مناطق الانتشار المنخفضة جدا يكون أحيانا 6-10 أضعاف ذلك. وفي مواجهة ما يبدو أنه عدد لا بأس به من "البلهارسيا سلبية البيض / إيجابية الديدان" وخاصة في مناطق الانتشار المنخفض جدا، تحار البرامج الوطنية NTD حول اختيار المبادئ التوجيهية والاستراتيجيات التي ينبغي أن تسن إذا ما أرادت المضي قدما نحو هدف التخلص من المرض. هناك حاجة ماسة للتقييم المستمر للفحوصات المعنية وفهم مساهمة مرض"البلهارسيا إيجابية البيض / سلبية الديدان"في الاعتلال الفردي وانتقال المرض في المجتمع. وهناك أيضا حاجة ماسة لوضع مبادئ توجيهية جديدة تقوم على استخدام هذه المقايسات الأكثر حساسية لبرامج NTD الوطنية التي ترغب في المضي قدما في الاستراتيجيات المصممة للقضاء على المرض.

Translated from English version into Arabic by Mahmoud Sami, through

TRANSLATORS WITHOUT BORDERS

血吸虫病比预想的更加普遍：对公共卫生目标、政策、战略、指南和干预计划意味着什么？

Daniel G. Colley, Tamara S. Andros and Carl H. Campbell, Jr.

摘要

使用已知的特异性测定法对三种主要血吸虫种感染（埃及血吸虫、曼氏血吸虫和日本血吸虫）情况进行绘图和诊断，但随着流行率下降或在低流行区，这种方法变得越来越不敏感。这成为实现血吸虫病消除目标的真正挑战，因为在血吸虫的繁殖阶段在钉螺体内，有利于其继续传播，只要甚至携带很少成虫的几个人就可以通过排泄物传播虫卵。基于感染者尿液中的成虫抗原检测（循环阴极抗原 – CCA；循环阳极抗原 - CAA）的新绘图工具高度敏感，并且 CAA 测定具有高度特异性。在这三种血吸虫病的低流行区，相比常规粪便检测法（Kato-Katz）或尿沉淀法可检测到的水平，新方法能检测出携带成虫的人数更多，有时在极低流行区可达 6 至 10 倍。特别是在极低流行区，“虫卵阴性/成虫阳性血吸虫病”患者似乎很多，国家 NTD 项目犹豫应该制定何种指南和策略才能实现消除血吸虫病的目标。有必要继续评估相关检测方法，并了解

“虫卵阴性/成虫阳性血吸虫病”在个体发病率和社区传播中的作用。同时，还迫切需要一种基于更敏感检测方法的新指南，用于国家 NTD 项目，以期推进血吸虫病消除战略。

Translated from English version into Chinese by Jin Chen, edited by Pin Yang

CHINESE CENTER FOR DISEASE CONTROL AND PREVENTION
NATIONAL INSTITUTE OF PARASITIC DISEASES

La schistosomiase est plus répandue qu'on ne le pensait : que cela implique-t-il en termes d'objectifs, de politiques, de stratégies, de directives et de programmes d'intervention en matière de santé publique?

Daniel G. Colley, Tamara S. Andros et Carl H. Campbell, Jr.

Résumé

Une cartographie et un diagnostic des infections regroupées par les trois principales espèces de schistosomes (*Schistosoma haematobium*, *S. mansoni* et *S. japonicum*) ont été réalisés avec des dosages qui reconnus pour leur précision, mais celle-ci est de plus en plus obsolète dans les zones ou elles sont en déclin ou avec une roccurence très faible. Il est de plus en plus difficile d'éliminer la schistosomiase parce que la partie multiplicative du cycle de vie des schistosomes, dans le vecteur d'escargot, favorisa la transmission continue aussi longtemps que de personnes n'auront toujours que peu de nombres passant les les œufs dans leurs déjections. Les nouveaux outils de cartographie basés sur la détection d'antigènes de vers (antigène circulant cathodique - CCA ; antigène circulant anodique - CAA) dans l'urine des personnes infectées sont très sensibles et le dosage de la CAA semble être extrêmement précis. L'utilisation de ces outils dans les zones de faible prévalence de ces trois espèces de schistosomes a démontré que plus de personnes sont porteuses de vers adultes que ne sont excrétés régulièrement d'œufs à un niveau détectable par le test habituellement utilisé des selles (Kato-Katz) ou par l'examen du sédiment urinaire. Dans les zones à faible prévalence, elles le sont par cinq à six fois plus. Face à ce qui semble être une population importante de « œuf-négatif / ver positif schistosomiase », surtout dans les zones à très faible prévalence, les programmes nationaux NTD ne savent pas très bien quelles lignes directrices et stratégies adopter s'ils décident de cibler l'élimination. Il y a un besoin critique d'évaluation continue des dosages concernés et de comprendre l'impact de cette condition « oeuf négatif / ver positif schistosomiase », condition à la fois sur la mortalité et la transmission dans la communauté. Il y a aussi un besoin urgent de nouvelles lignes directrices fondées sur l'utilisation de ces tests plus sensibles pour les programmes nationaux NTD qui souhaitent mettre en avant des stratégies d'élimination.

Translated from English version into French by Sandrine Savarit, through

TRANSLATORS
WITHOUT BORDERS

Шистосомоз встречается куда чаще, чем считалось ранее. Что это значит для целей государственного здравоохранения, политики, стратегий, рекомендаций и программ реализации мероприятий?

Даниэль Г. Колли, Тамара С. Андрос и Карл Х. Кэмпбелл-младший

Аннотация

Отображение и диагностика инфекций трех основных видов шистосом (*Schistosoma haematobium*, *С. mansoni* и *С. japonicum*) были осуществлены путем проведения анализов, которые являются конкретными, но демонстрирую все меньшую и меньшую чувствительность по мере снижения распространенности инфекции или в районах, в которых уже зафиксирован низкий уровень распространенности инфекции. Это представляет собой значительную трудность при достижении цели, связанной с ликвидацией шистосомоза, поскольку часть жизненного цикла шистосом, связанная с их размножением среди улиток, способствует непрерывной передаче инфекции до тех пор, пока хотя бы несколько человек являются носителями небольшого количество паразитов, яйца которых распространяются вместе с человеческими экскрементами. Новые инструменты проектирования, основанные на обнаружении антигенов червя (циркулирующего катодного антигена - ССА; циркулирующего анодного антигена - САА) в моче инфицированных, являются весьма чувствительными, в то время как анализ САА показывает достаточно конкретные результаты. С помощью этих средств, использованных в районах с низкой распространенностью всех этих трех видов шистосом, удалось доказать, что все больше людей являются носителями взрослых червей, которые регулярно откладывают яйца на уровне, обнаруживаемом с помощью обычного анализа кала (методом Като-Катца) или оседания мочи. В районах с очень низким уровнем распространенности этот показатель может быть превышен в 6-10 раз. Столкнувшись с, казалось бы, значительной популяцией "шистосом, для которых характерно отсутствие яиц и наличие червей", особенно в районах с очень низким уровнем распространенности, руководители национальных программы NTD испытывают трудности при выборе подлежащих реализации стратегий и инструкций в случае необходимости достижения цели ликвидации выявленной инфекции. Существует острая необходимость дальнейшей оценки соответствующих анализов и осознания той роли, которую играет "шистосомоз, для которого характерно отсутствие яиц и наличие червей", в определении уровня заболеваемости и передачи инфекции в сообществе. Существует также острая необходимость в новых руководящих принципов, основанных на использовании этих более чувствительных анализов для национальных программ NTD, которые хотят двигаться вперед стратегий, направленных на устранение.

Translated from English version into Russian by Irina Zayonchkovskaya, through

TRANSLATORS
WITHOUT BORDERS

La esquistosomiasis es más frecuente de lo que se pensaba: ¿qué significa para los objetivos de la salud pública, las políticas, estrategias, directrices y los programas de intervención?

Daniel G. Colley, Tamara S. Andros y Carl H. Campbell, Jr.

Resumen

El mapeo y diagnóstico de las infecciones por las tres principales especies de esquistosomas (*Schistosoma haematobium*, *S. mansoni* y *S. japonicum*) se ha hecho con ensayos que se saben específicos pero que se tornan cada vez más insensibles conforme disminuye la prevalencia o en áreas con ya baja prevalencia de la infección. Esto se convierte en un verdadero desafío para alcanzar la meta de eliminación de la enfermedad, ya que la porción multiplicativa del ciclo de vida de los esquistosomas, en el vector de caracol, favorece la transmisión continuada, siempre y cuando al menos algunas personas mantengan números bajos de gusanos que depositen huevos en sus excrementos. Nuevas herramientas de mapeo basadas en la detección de antígenos de gusano (antígeno catódico circulante - CCA; antígeno anódico circulante - CAA) en la orina de las personas infectadas son muy sensibles y se ha reportado que el ensayo de CAA es altamente específico. El uso de estas herramientas en áreas de baja prevalencia de las tres especies de esquistosomas ha demostrado que hay más personas que albergan gusanos adultos de las que regularmente excretan huevos a un nivel detectable por los análisis de heces normalmente utilizados (Kato-Katz) o por sedimentación de la orina. En las zonas de muy baja prevalencia, esta diferencia se puede multiplicar entre 6 y 10 veces. Frente a lo que parece ser una población considerable de "esquistosomiasis huevo-negativa / gusano-positiva", sobre todo en zonas de muy baja prevalencia, los programas nacionales de ETD son vagos en cuanto a las directrices y estrategias que deben poner en práctica si han de proceder hacia una meta de eliminación. Hay una necesidad crítica de evaluación continua de los ensayos involucrados y comprensión de la contribución de esta condición de "esquistosomiasis huevo-negativa / gusano-positiva" a tanto la morbilidad individual como la transmisión en la comunidad. También hay una necesidad crítica de nuevas directrices basadas en el uso de estos ensayos más sensibles para aquellos programas nacionales de ETD que deseen avanzar hacia estrategias diseñadas para la eliminación.

Translated from English version into Spanish by Aldana Gómez Ríos, through

TRANSLATORS
WITHOUT BORDERS
